# Surgical evolution in the treatment of mandibular condyle fractures

**DOI:** 10.1186/s12893-015-0001-9

**Published:** 2015-03-08

**Authors:** Evaristo Belli, Gianmauro Liberatore, Mici Elidon, Giovanni Dell’Aversana Orabona, Pasquale Piombino, Fabio Maglitto, Luciano Catalfamo, Giacomo De Riu

**Affiliations:** Maxillofacial Surgery Department, Sant’Andrea Hospital, “Sapienza” University of Rome, Rome, Italy; Maxillofacial Surgery Department, Azienda Ospedaliera Universitaria Pisana of Pisa, Pisa, Italy; Maxillofacial Surgery Department, University of study of Messina, Messina, Italy; Maxillofacial Surgery Department, Federico II University of Naples, Naples, Italy; Maxillofacial Surgery Department and Dentistry Department, University Hospital of Sassari, Sassari, Italy

**Keywords:** Mandibular condyle fracture, Mandible fracture, Endoscopic surgery, Temporal mandibular joint

## Abstract

**Background:**

In Literature fractures of the mandible that involve the condyle ranges from 20% to 35% and various possible surgical options are described according to the varying pathological situations. Up to the present, numerous techniques have been used for the surgical treatment of condylar fractures. In this article we are proposing the combination of two surgical techniques as therapy for extra-capsular condylar fractures with dislocation.

**Methods:**

From June 2003 to July 2007 30 patients were treated for condylar fractures with the application of a Rigid External Fixator under endoscopic assistance. This method includes a surgical reduction of the fracture with the aid of an endoscope, performing a transcutaneous insertion of a Rigid External Fixator to stabilize the fracture.

**Results:**

Out of the total number of patients, 28 reached an optimal result without the need for temporary immobilization of the temporal mandibular joint and pre-auricular cutaneous access, thanks to the decisive aid of the video-endoscope.

**Conclusions:**

The endoscope allows perfect control over both the positioning of the external fixator and the surgical reduction, restoring the normal movement of the mandible with a return to full anatomical functioning of the temporo-mandibular joint. This approach avoids possible damages to the facial nerve branches. The rigid external fixation system is better than an internal one, because it is less restrictive in precise anatomical reduction, since with an REF the condylar fragment is kept in the correct anatomical position but is not obliged to maintain that exact position, and therefore it is possible to carry out all the repair mechanisms listed above. Endoscopic assistance allows a good positioning control of the REF although the endoscopy permits an optimal control of the condylemeniscal complex mobility after REF application.

## Background

In the International Literature, fractures of the mandible that involve the condyle ranges from 20% to 35% [1]. The condyle represents a structural weak point in the mandibular skeleton because of its shape and the slenderness of its neck, and sometimes its being fractured avoids more serious consequences such as fractures of the base of the skull which can traumatically interrupt propulsive strength [2]. The position of the fracture is related not only to the location and severity of the trauma but also to the position and action of the masticatory muscles as well as the presence of dental elements. Various surgical options are possible according to the varying pathological situations. Among cases of intracapsular fracture in which the most advisable treatment to date ranges from an approach to preserve the function to an almost compulsory surgical reduction in cases of bilateral condylar dislocation due to panfacial trauma, there are several possibilities and options which have inspired differing attitudes on the part of various authors, particularly as regards indications for “open” surgical therapy.

Up to the present, numerous techniques have been used for the surgical treatment of condylar fractures: from osteosynthesis using metal wire, to mini-systems with rigid internal fixing, or various types of pin inserted through cutaneous approaches – whether pre-auricular, sub-mandible, trans-parotid or the use of a system of rigid external fixing after an open reduction through preauricular access, introduced in Italy in 1990 by Cascone [3]. Later on, in 1999, was presented a surgical technique to reposition extra-capsular condylar fractures by an endoral approach under video-endoscopic control featuring a rigid inter-maxillary blockage. Belli in 2007 introduced the navigation system combined to endoscopy for condylar approaches [4]. In this work we are proposing a combination of these two surgical techniques, modifying them and thus obtaining two fundamental advantages with respect to the individual methods: an inter-maxillary blockage – so psychologically “bothersome” for the patient – is no longer necessary, and it becomes possible to avoid a pre-auricular cutaneous incision which can produce scarring, scarcely visible though it may be.

## Methods

**Figure 1:**
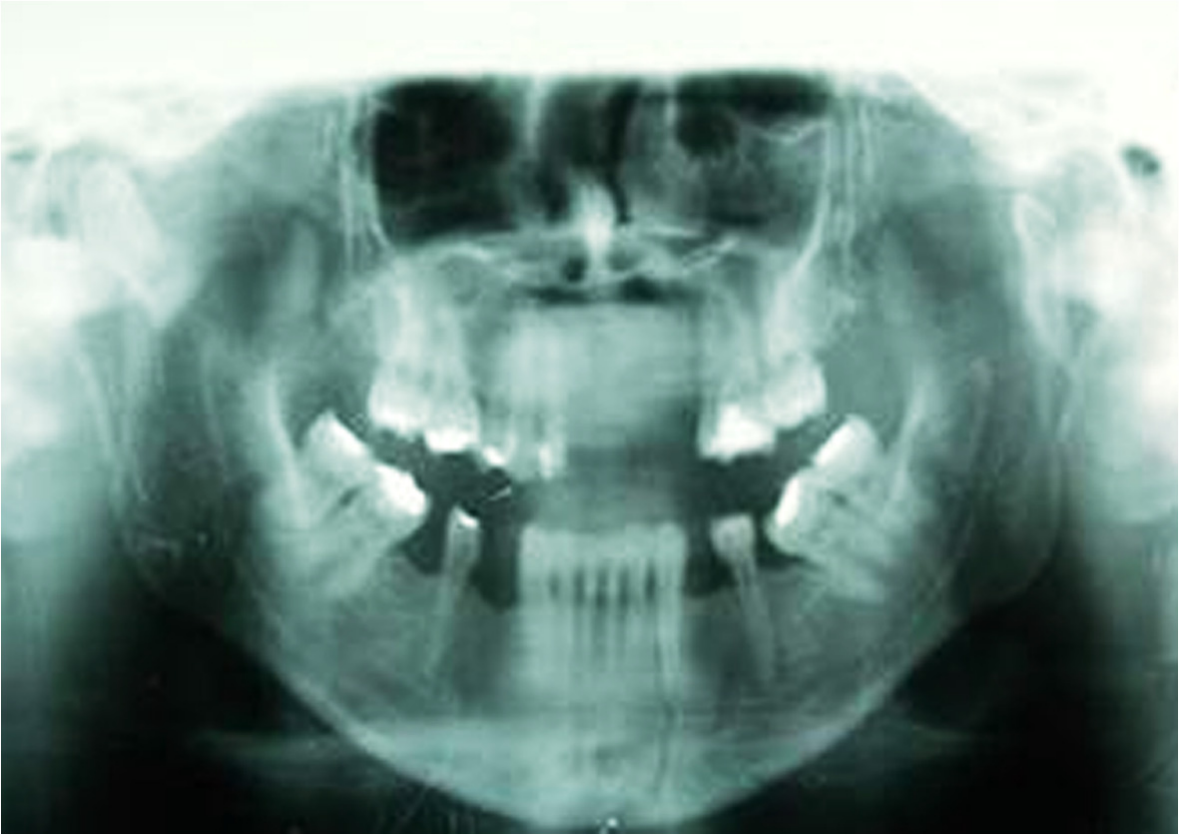
**Pre operative Orthopantomograph.**

**Figure 2:**
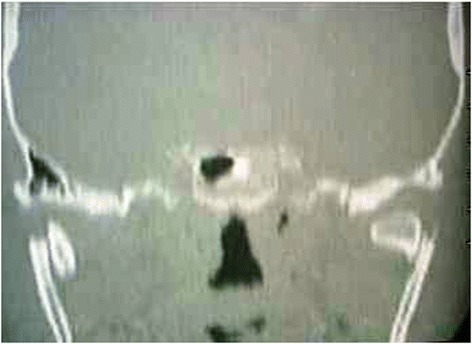
**CT scan that indicat surgical treatment.**

From June 2003 to July 2007, 32 patients with mandibular condyle fractures (including 5 with bilateral fractures) underwent surgical treatment (Table 1). Ages ranged from 10 to 55 years and the sexes were represented by 7 females and 25 males. Clinical diagnosis was always accompanied by a radiological examination of the mandible using an Orthopantomograph (Figure 1), plus a CT scan of the mandible whenever the standard radiograph indicated surgical treatment (Figure 2).

**Table 1:** **Patients treated**

Total patients treated	32
Intermaxillary Fixation	4
Aditional procedures	5
Re operated patients	2
Satisfactory results	23

**Figure 3:**
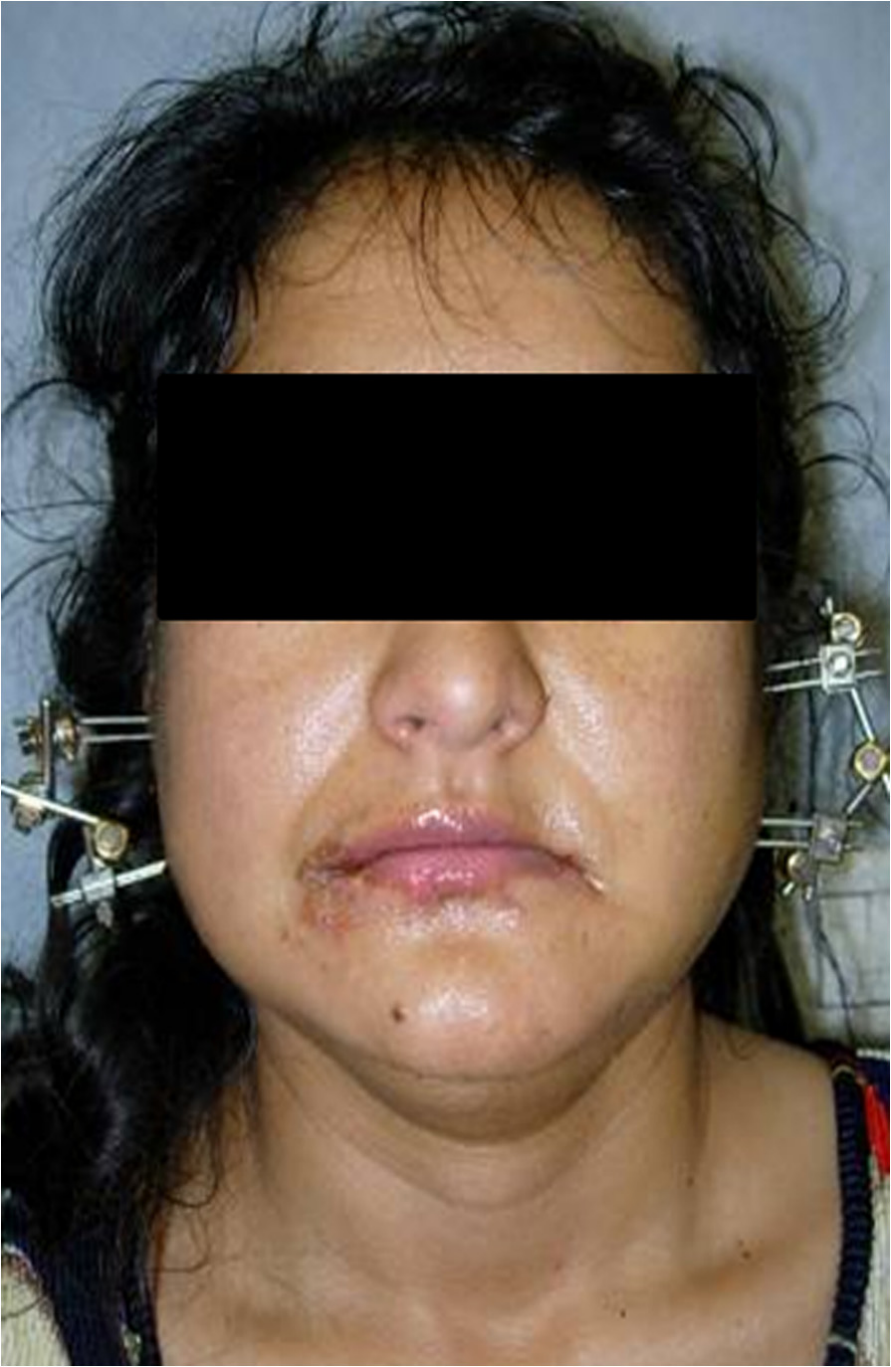
**Rigid external fixation system produced by the Stryker company.**

**Figure 4:**
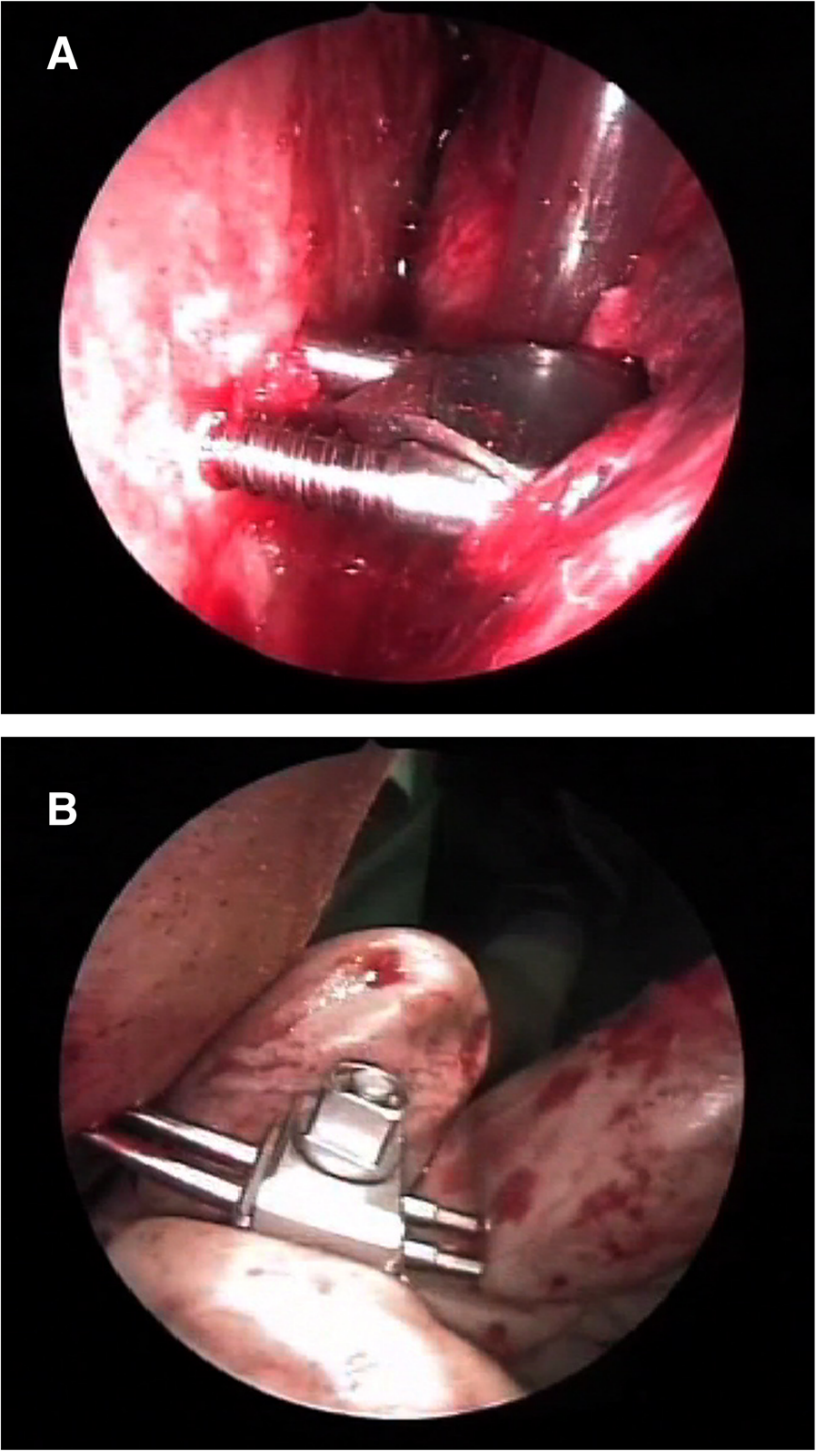
**Endoscopic view of pins inserted into the condyle stump (a) and into the ramus (b).**

**Figure 5:**
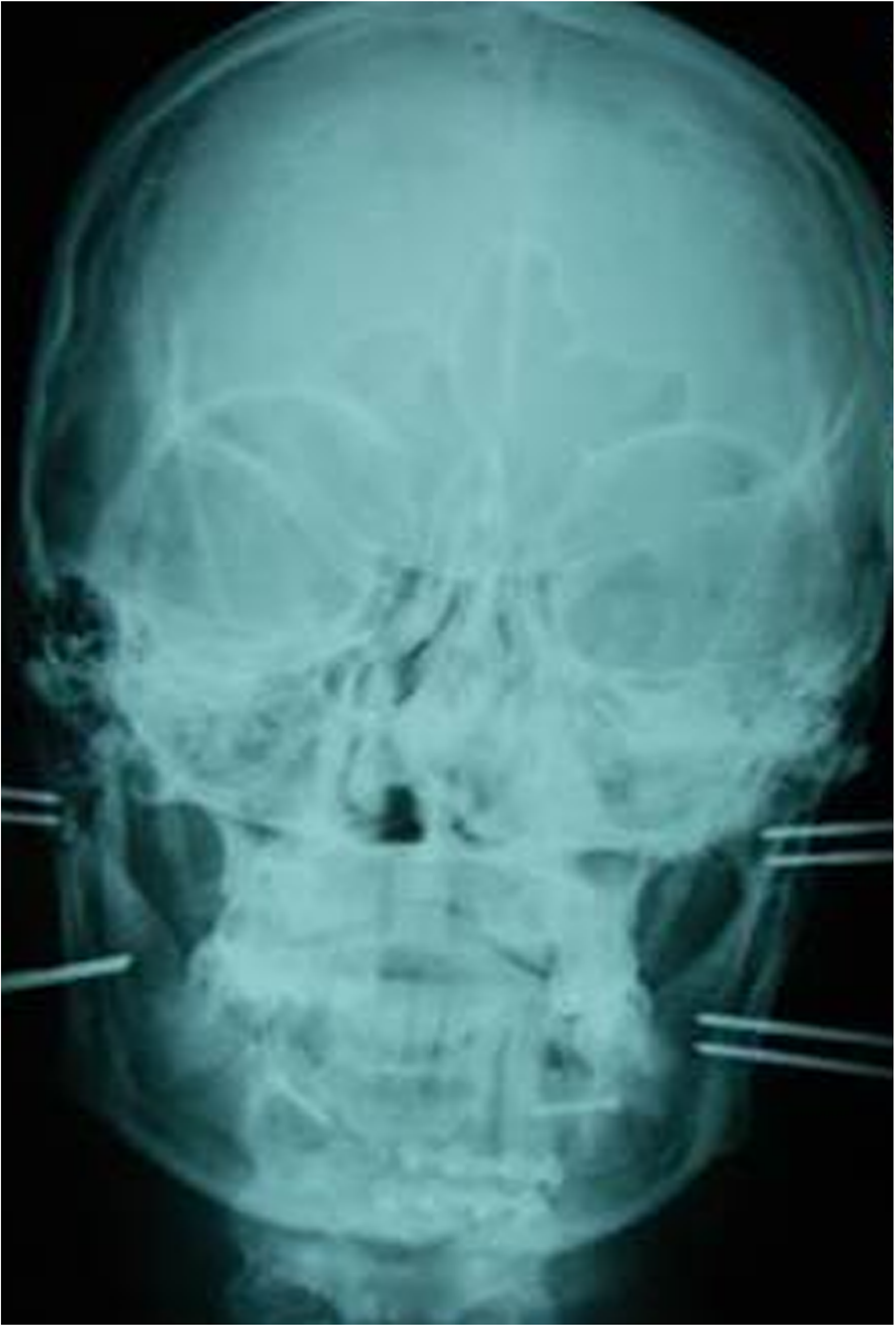
**Post surgical CT scan.**

The technique proposed envisages tracing the path of the condyle and then repositioning it under video-endoscopic control, by an endoral approach through an incision at the level of the homolateral retromolar trigone, as well as opening the jaw below the periosteum and the posterior border of the mandible to find the fracture focus. Endoscopes with 0°, 30°, 45° and 70° angulations were used according to the type of surgery, with the aid of a Xenon light source [5]. Traditional surgical equipment was used for the open surgical treatment of maxillary-facial traumas in combination with the kind of angled aspirator used in endoscopic nasal surgery. Once the fracture had been reduced, it was stabilized by using a rigid external fixation system produced by the Stryker company (Figure 3). This system is called Hoffmann II Micro Stryker HowMedica and consists of a series of pins, clamps and connecting rods in light and ultra-light biocompatible material which were used in conjunction with yet another system for mandibular bone distraction, produced by Leibinger-Stryker and called Multi-guide II Mandibular Distraction Device. By using two systems readily available on the market, a mixed system was created which is adaptable to any type of fracture. The Rigid External Fixator (REF) consists of a series of pins which are introduced through atraumatic subcutaneous incisions at a pretragic level until the fractured stump of the condyle is reached, while other two pins are inserted near the corner or into the ramus of the mandible, again through atraumatic subcutaneous incisions (Figure 4a and b). The instrumental examinations included CT scan (Figure 5) and EKG examination which shows the functioning of the mandible on the computer. Unfortunately it was not possible to carry out this examination in all cases, nor was it possible to do so during the diagnostic, pre-operative phase. However, in our opinion, this examination becomes fundamental in remote check-ups since it is non-invasive and repeatable every time it is deemed opportune to compare the clinical evolution of the mandibular movements. As of mid-2006, thanks to collaboration with the Department of Orthognathology and Gnathology in our hospitals, we have begun to offer a phase of post-surgery rehabilitation to all patients treated with our surgical method, featuring variable cycles of functional therapy that use mandibular activators, such as the Balters’ Bionator.

## Results

Only in 4 cases was it necessary to position an inter-maxillary blockage for seven days because of the contemporary presence of contralateral intracapsular condylar fracture treated with REF. Five patients requested an additional procedure with a pre-auricular cutaneous approach as described in the original technique proposed by. Out of the total number of patients, 28 reached an optimal result without the need for temporary immobilization of the temporal mandibular joint and pre-auricular cutaneous access, thanks to the decisive aid of the video-endoscope. Out of a total of 32 patients, 4 out of 5 who presented complications did not satisfactorily resolve their problem and of these, 2 underwent new operations to resolve their fractures. The main problems experienced by these 5 patients arose from an inadequate repositioning of the fractured piece with lateral/controlateral movements which were much reduced compared to normal. Nonetheless, the opening of the mouth was satisfactory and the patients’ subjective symptoms did not include pain but only the sensation of a functional impediment in lateral movements; on opening the mouth was a persistent degree of both lateral and protrusive deviation.

## Discussion

Fractures of the condyle are still today the subject of much discussion, especially as regards standardizing the therapy, due to the wide variety of forms this may take, and because of the numerous therapeutic methods available.

The necessity for a simple classificatory criterion is of fundamental importance to correctly apply any therapy, which must necessarily take into account parameters such as the age of the patient, the intra- or extra-capsular location of the fracture, whether it is mono- or bi-lateral, the kind of dislocation of the stumps and the presence or absence of luxation of the condylar head from the glenoid cavity.

Also the age of the patient governs the type of therapeutic treatment. During the years of growth some authors have found a greater capacity for morphofunctional recovery of the fractured condyle in comparison with adult patients. Thus the therapeutic approach can vary not only according to the type of fracture, but also the type of patient. The two main therapeutic directions envisage on the one hand orthopaedic-functional treatment and on the other surgical treatment. Orthopaedic-functional therapy remains the most commonly used by various authors, permitting as it does an optimal functional recovery. Here it must be underlined that an inter-maxillary blockage determines two main problems; as well as immediate morphofunctional limitations due to the complex nature of the temporal-mandibular joint, there are often further psychological problems for the patient. However, despite these, the bibliography is lavish in its support of the efficacy of this particular therapeutic approach. The orthopaedic-functional approach has always been practiced in condylar fractures in the pediatric age, and for intra- and extra-capsular fractures without serious condylar dislocation in adult subjects [6-8]. Delaire, in 1975, held functional therapy to be necessary with early mobilization in cases of dislocated subcondylar fractures, whether high or low, and in every type of fracture of the condylar head. This treatment is performed to avoid tardive complications such fibrosis and ankilosis [9,10]. The results described are, on the whole, positive. In fact, according to Delaire they are particularly encouraging in young children. Takenoshita in comparing 16 cases of condylar fractures treated surgically with 20 cases treated in a conservative manner with a minimum follow-up of 2 years, found no important functional differences between the two groups, even if in the first group there were fractures with notable luxation and displacements [11]. Surgical therapy is generally adopted in cases where it is not possible to make use of a conservative treatment, or where this would not guarantee an adequate recovery *ad integrum*. In 1983, the various indications for surgical treatment were schematized by Zide and Kent in absolute and relative terms. In the latter case, the possibility of surgical treatment is particularly important in as much as such fractures provoke a reduction in the posterior facial height which must necessarily be recovered through surgery, to give an adequate guide parameter for successive threedimensional reconstruction of the face [12,13].

Various authors maintain that surgical therapy is indicated in cases of mono-condylar fractures in adults or adolescents, not only where it is impossible to achieve normal occlusion, but also where there is noteworthy dislocation, with an angle of the small fragment greater than 45° [4] or simply where the condylar head has luxated from the glenoid cavity [14]. In fact, in these cases, conservative therapy, whilst assuring good dental occlusion in general terms, often does not allow complete recovery of the mandibular movements [15]. Moreover, in the opinion of other authors too, it is difficult to achieve completely satisfactory results from both an aesthetic viewpoint (due to the reduction in height of the ramus) and a gnathological standpoint because of the frequent presence of pre-contact during mandibular movements. Very recently, the direction of the treatment to resolve the greatest possible number of problems linked to fractures of the mandibular condyle, is fast tending towards a surgical approach, thanks not only to new physio-biomechanical acquisitions of the complex temporal-mandibular joint, but also to the development of new surgical techniques such as REFs which allow an optimal adaptation of the fractured fragments without the need for inter-maxillary blockage and the resultant immobilisation of the temporal-mandibular joint. Broadly speaking, the choice of surgical technique is conditioned by various factors such as :the focus of the fracture,the position of the condyle,the time elapsed from the traumatism,the extent of local oedema the type of surgical.

Until only a few years ago, the concept reigned that surgical access had to be such as to allow the most direct approach possible to the dislocated condyle stump.

## Conclusions

Analysing the evolution of thinking on therapeutic approaches proposed over the last few years, we began to consider condylar fractures a more and more delicate problem, and the therapeutic approach the preferable choice, since it seems to us to be the one which aims at obtaining a morphofunctional recovery leading to a situation which is the most similar to that before the trauma. In this vision, the targeted surgical approach is gathering consensus, but the originality of the method we have introduced lies above all in trying to avoid immobilising the complex temporalmandibular joint system, in making external cutaneous incisions to reduce to a minimum both scarring and lesions of certain branches of the facial nerve, plus the use of a rigid external fixation system (REF), already used extensively in the recovery of fractures in other areas of the body by our orthopaedic colleagues. Endoscopic assistance allows a good positioning control of the REF although the endoscopy permits an optimal control of the condyle-meniscal complex mobility after REF application. Endoscopy nowadays is commonly used in maxillofacial surgery, such as oncology, trauma [16-21]. The main indications for using our method are isolated mono- or bi-condylar extra-capsular dislocated fractures, or for other fractures of the mandible which require rigid internal containment, and which present notable functional limitation. In our opinion, the rigid external fixation system is better than an internal one, because it is less restrictive in precise anatomical reduction, since with an REF the condylar fragment is kept in the correct anatomical position but is not obliged to maintain that exact position, and therefore it is possible to carry out all the repair mechanisms listed above. This method can also be used for paediatric patients without producing anti-aesthetic scars or fibrosis from excessive deperiostation, and, furthermore, the rigid fixator is rapidly removed in the clinic without interfering with skeletal growth in infant patients. Aesthetical results must be considered in facial trauma management [19]. Reduction in adult patients must aim at precise anatomical recovery in order to avoid generating functional alterations of the complex temporal-mandibular joint system and thus cause a TMA. The application of Rigid External Fixation can be performed by intraoral approach under endoscopic control and offers good results. Although sometimes in panfacial fractures or in pre existing scars we have to perform pre auricular incision.

### Ethics statement

All patients granted written specific consent for all video, photographs and personal data to be used in every medical publications, journal, textbook and electronic publications. The study was conducted in accordance with the ethical principles provided by the Declaration of Helsinki and the principles of good clinical practice. Study design, inclusion and exclusion criteria and treatment protocol were reviewed and approved by a council of senior specialists at the Maxillofacial Surgery Department, Sant’Andrea Hospital of Rome, Italy.
