# Erratum to: Surgical evolution in the treatment of mandibular condyle fractures

**DOI:** 10.1186/s12893-015-0042-0

**Published:** 2015-06-04

**Authors:** Evaristo Belli, Gianmauro Liberatore, Elidon Mici, Giovanni Dell’Aversana Orabona, Pasquale Piombino, Fabio Maglitto, Luciano Catalfamo, Giacomo De Riu

**Affiliations:** Maxillofacial Surgery Department, Sant’Andrea Hospital, “Sapienza” University of Rome, Rome, Italy; Maxillofacial Surgery Department, Azienda Ospedaliera Universitaria Pisana of Pisa, Pisa, Italy; Maxillofacial Surgery Department, University of study of Messina, Messina, Italy; Maxillofacial Surgery Department, Federico II University of Naples, Naples, Italy; Maxillofacial Surgery Department and Dentistry Department, University Hospital of Sassari, Sassari, Italy

## Correction

There was an error with one Author name (Number 3). Name and surname were inverted. The correct one is: NAME: Elidon and SURNAME: Mici.

